# Developing and Evaluating the Greenness of a Reliable, All-in-One Thin-Film Microextraction Protocol for Determining Fentanyl, Methadone, and Zolpidem in Plasma, Urine, and Oral Fluid

**DOI:** 10.3390/molecules29020335

**Published:** 2024-01-09

**Authors:** Krzysztof Goryński, Łukasz Sobczak, Dominika Kołodziej

**Affiliations:** 1 Faculty of Chemical Technology and Engineering, Bydgoszcz University of Science and Technology, Seminaryjna 3, 85-326 Bydgoszcz, Poland; 2 Faculty of Pharmacy, Nicolaus Copernicus University in Toruń, Jurasza 2, 85-089 Bydgoszcz, Poland

**Keywords:** solid-phase microextraction, oral fluid, urine, blood, opioids, zolpidem, fentanyl, methadone, overdosing, LC-MS/MS

## Abstract

This paper proposes an all-in-one microextraction-based protocol capable of determining and quantifying fentanyl, methadone, and zolpidem in plasma, urine, and saliva at concentrations below those required by international regulatory organizations. A homemade thin-film microextraction device featuring an octyl–cyanopropyl stationary phase was coupled with LC-MS/MS. The proposed method was developed and validated according to FDA criteria, providing extraction efficiency values ranging from 26.7% to 76.2% with no significant matrix effects (2.6% to 15.5% signal suppression). The developed protocol provided low limits of quantification (mostly equal to 1 ng mL^−1^) and good reproducibility (intra- and inter-day RSDs of less than 9.6% and 12.0%, respectively) and accuracy (89% to 104% of the test concentration). An assessment of the protocol’s environmental impact indicated that attention must be devoted to eliminating the use of toxic reagents and developing its capability for in situ sampling and in-field analysis using portable instruments. The proposed TFME-based protocol provides clinical laboratories with a versatile, one-step tool that enables the simultaneous monitoring of fentanyl, methadone, and zolpidem using the most popular biological matrices.

## 1. Introduction

Potent prescription-only medications (POMs) are increasingly being misused for various non-medical purposes and have become the leading cause of overdose deaths worldwide. According to the World Health Organization (WHO) [1], nearly 600,000 deaths around the world can be attributed to drug abuse each year, with 80% being related to opioid abuse and approximately 25% due to opioid overdose. Since 2016, fentanyl, a powerful synthetic opioid (50–100 times stronger than morphine) used as a pain reliever and anesthetic, and its analogs have been the main contributors to a spike in opioid overdose deaths, largely due to increased availability via illegal manufacturing and distribution. There is also evidence that drug dealers may be adding fentanyl to other products (e.g., heroin) to increase their potency, selling counterfeit opioid prescriptions through illicit channels, and distributing tablets that look like authentic fentanyl. As a result, many users who test positive for fentanyl and its analogs do not realize that they have used synthetic opioids, thus increasing their risk of overdose. The Centers for Disease Control (CDC) has conducted numerous studies and published the resultant data to broaden understanding of the opioid overdose epidemic [2]. As shown in Figure S1, over six times as many people died of drug overdoses in 2021 compared to 1999, with synthetic opioids such as fentanyl linked to >80% of these deaths. This increase spiked dramatically in the third wave, which began in 2013.

The WHO recommends a range of treatment options for opioid dependence, including opioid agonist maintenance treatment (using medicines like buprenorphine and methadone), which evidence has shown to be the most effective and economical treatment [1,3]. The US Food and Drug Administration (FDA) has approved the use of methadone, a synthetic opioid, in the treatment of opioid use disorder (OUD) due to its considerable efficacy and ability to save lives [3]. Thus, methadone use has also increased substantially over the past decade, mostly due to OUD-related applications. Unfortunately, methadone use has a high likelihood of resulting in overdose due to the lack of a ceiling effect; that is, people can graduate from an initial dose of 10–30 mg/day to potentially toxic levels (e.g., 80–160 mg/day) over the course of only a few days [4]. Despite being considered a safe alternative to opioids, the excessive use of methadone can result in severe physical and mental injury or even death [4].

In 2016, the FDA issued a black box warning regarding the potential risk of respiratory depression following the combined use of opioids and benzodiazepines or other CNS depressants, including sleep drugs (e.g., Z-drugs and barbiturates) [5]. Despite this warning, the use of Z-drugs (e.g., zolpidem, zopiclone, and zaleplon) remains widespread among prescription opioid users, regardless of the potential hazards. Although Z-drugs are commonly considered safer compared to benzodiazepines due to their lower susceptibility to abuse, addiction, and tolerance, evidence for zolpidem abuse, dependency, and withdrawal at therapeutic and supratherapeutic doses has emerged over the last few years [6].

The rise in the number of drugs abused worldwide, along with polyconsumption and resultant overdoses, has become a serious public health problem [1,2,4]. As such, many regulatory and health emergency organizations (e.g., The European Workplace Drug Testing Society (EWDTS), the Substance Abuse and Mental Health Services Administration (SAMHSA), the United Nations Office on Drugs and Crime (UNODC)), in a combined effort with the WHO, FDA, and World Anti-Doping Agency (WADA), routinely employ sensitive and specific analytical assays to identify and quantify drugs and their products in biological samples.

For many years, primary screening analyses exclusively consisted of immunoassay techniques; as such, methods are fast, cost-effective, and usually employ small sample volumes and automated systems. However, immunoassays lack specificity; that is, they cannot differentiate drugs from the same class [7]. Immunoassays are unable to detect several synthetic compounds, and the occurrence of cross-reactions can result in false-positive rates ranging between 9 and 53%, depending on the device and the target drug [8]. Another drawback is that lateral flow immunoassay tests tend to have short shelf lives due to the use of bioreceptors. Consequently, it is necessary to perform additional confirmatory analyses using chromatographic methods coupled with mass spectrometry (LC-MS/MS), as this approach provides comprehensive, sensitive, and specific analysis using low sample volumes.

It is difficult to detect and quantify drugs of abuse in biological matrices—even for regulatory authorities and analysts—due to their high complexity, as well as the wide range of compounds that must often be monitored, the quantity of biological samples under analysis, and the complications related to the time of analysis. Thus, the main challenge for scientists is to properly define the final protocol that will be used to analyze a biological sample, including the sample preparation technique and the chromatographic method. Several studies have analyzed fentanyl, methadone, and zolpidem in whole blood and its derivatives, serum, and plasma [9,10]. The higher frequency is also noted for samples of urine and most recently oral fluid and dry samples (dried blood spot and dried urine spot) [11,12].

The preparation of complex biological samples prior to LC-MS/MS analysis is critical, as proper sample preparation can ensure the accurate identification and quantification of drugs. Furthermore, selecting the optimal sample preparation method is also integral to ensuring the resultant protocol is reliable and reproducible; for a discussion of the specific advantages and disadvantages of the different sample preparation microextraction-based protocols for drugs of abuse please see the reviews published recently [13,14,15,16]. Most of the sample preparation techniques presented in the last few years have been rooted in the principles of green chemistry, especially the technique’s miniaturization. Another important aspect worth exploring is method automation, as this feature minimizes the need for human sample handling—and thus, the chance of errors and sample manipulation—and enables faster analysis and results regarding the presence and concentration of drugs in a given sample [15]. When choosing a sample preparation method, it is also important to give special attention to the efficiency and reproducibility of the extraction, the sensitivity of the analysis, the required amounts of solvent and sample, and the cost and time of analysis [13,15]. Recently, several articles have confirmed the interest in many different microextraction-based methods for the analysis of drugs of abuse [17,18,19]. A recent paper presents dispersive pipette extraction (DPX) [17] using only 20 μL of sample with LOD starting from 0.4 ng mL^−1^. Another interesting article [18] described the use of 96-well liquid solution microextraction (parallel artificial liquid membrane extraction, PALME) demonstrating low cost, less than 1€ per sample, high sample throughput, and using only 4 μL of organic solvent for the supported liquid membrane. Another interesting future technique combining microsampling and microextraction was presented by Ask et al. [19]. In recent years, solid-phase microextraction (SPME) has been widely recognized as a valuable sample preparation method for the simultaneous analysis of drugs of abuse in complex matrices, including urine, plasma, blood, and oral fluid samples [20,21,22]. However, the selection of the SPME extraction phase depends on the applied chromatographic technique, and for users of LC-MS methods, commercially available SPME-LC coatings are limited to a few types of sorbents, with C18 being the most popular distributed by Sigma-Aldrich. At the time of this study’s publication, researchers using LC-MS have only demonstrated the utility of commercially available C18 SPME fibers or homemade blades (i.e., thin-film microextraction (TFME)) exploiting one type of stationary phase (e.g., C18, DVB, HLB, etc.) for the analysis of drugs of abuse [20,21,22] from one type of matrix (mainly plasma, urine, or oral fluid) or presenting analysis from multiple biological matrices, e.g., plasma and urine [23] or urine and oral fluid [24]. However, later studies [23,24] require a few modifications to the protocol (pH adjustments; different extraction volumes; different solvents; etc.) depending on the matrices applied.

In this paper, we present a fully validated and reliable all-in-one protocol based on homemade TFME devices coated with a stationary phase comprising an octyl–cyanopropyl mixture. The developed protocol is then applied for the determination and quantification of fentanyl, methadone, and zolpidem in every tested type of matrix, plasma, urine, and oral fluid at concentrations below those required by international organizations such as the EWDTS, SAMHSA, and the WADA. At the same time, criteria outlined by the FDA were rigorously tested during method validation. Finally, the developed protocol’s greenness was thoroughly evaluated against several existing green metrics [25,26,27,28].

## 2. Results and Discussion

### 2.1. Method Development

The goal of this work was to develop a practical, high-throughput microextraction-based sample preparation protocol that can be coupled with LC-MS/MS to measure fentanyl, methadone, and zolpidem in plasma, urine, and oral fluid samples. The LC-MS/MS method has been previously optimized and successfully applied for the separation of the same solutes in prior studies conducted by our group [29,30]. It should be noted that the sample solvent was diluted using an autosampler prior to LC injection to prevent the deterioration of the peak shapes, which tends to occur when the sample solvent’s elution strength is higher compared to the mobile phase. For each sample, 0.8 µL of extract (in acetonitrile/water/formic acid (80/19.9/0.1, *v*/*v*/*v*)) was diluted with 3.2 µL of water for a total injection volume of 4.0 µL. The final chromatographic parameters and the mass spectrometry settings are described in Section 3.6 (HPLC-MS/MS Method), while the chromatograms and retention times with precursor–product ion transitions are presented in the Supplementary Materials (Figure S2 and in Table S1, respectively). The LC-MS/MS method’s reproducibility was tested via multiple injections on different days, with RSDs of 4.8%, 4.8%, and 5.8% being obtained for fentanyl, methadone, and zolpidem, respectively.

**Figure 1:**
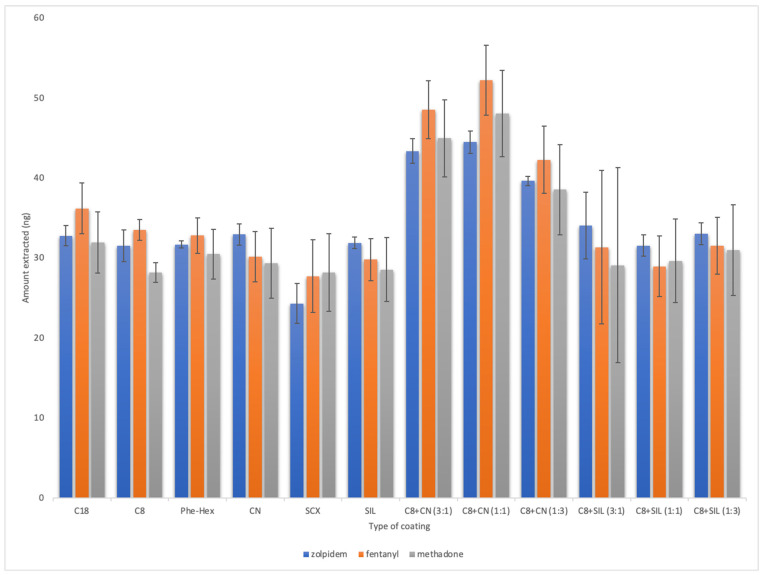
Comparison of different types of coatings (*n* = 3, extract from PBS, desorption solvent can/water/FA 80:19.9:0.1 *v*/*v*/*v*; desorption and extraction times were 150 and 120 min, respectively, in all cases).

**Figure 2:**
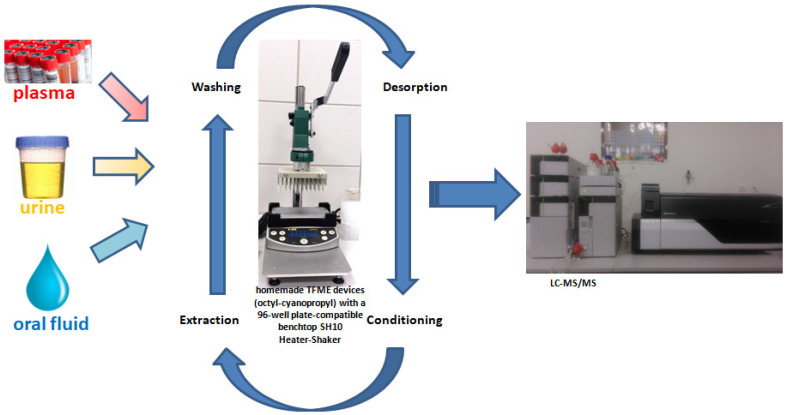
Schematic presentation of the all-in-one thin-film microextraction protocol coupled with LC-MS technique. Parameters used with TFME protocol summarized in Figure 3.

**Figure 3:**
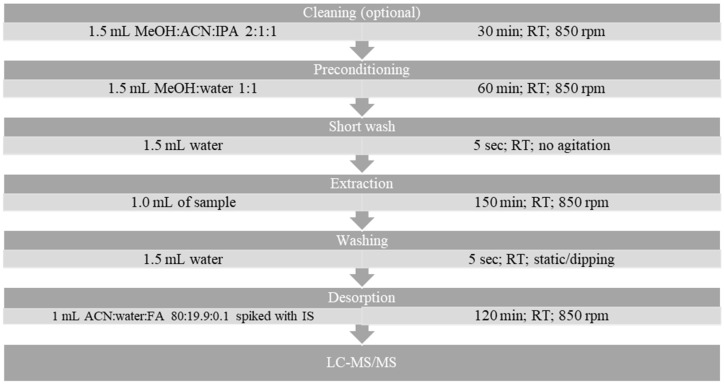
Parameters used in final protocol.

The first variable studied during the development of the sample preparation method was the extraction phase, as optimizing this component can ensure adequate extraction efficiency. To this end, unbounded silica and five types of silica particles bonded with functional groups were tested. Additionally, mixed compositions combining non-polar C8 particles with polar SIL or less nonpolar CN-type particles were prepared at ratios of 3:1, 1:1, and 1:3 (m/m). The results obtained for the extraction of fentanyl, methadone, and zolpidem using each sorbent are shown in Figure 1. As can be seen, the sorbents composed of different ratios of C8 and CN particles provided the highest extraction efficiencies, likely because the combination of interactions between the analytes and the extraction phase ligands (e.g., π-π, dipole–dipole, and hydrophobic-type interactions) facilitated their extraction from the sample. The final extraction phase was selected based on an evaluation of factors such as extraction efficiency and reproducibility, with the coating comprising C8 and CN at a ratio of 1:1 ultimately providing the best results. In developing SPME-based methods, it is also highly important to consider other important parameters, such as the desorption solvent, the extraction and desorption times, and the temperature and agitation speed used during extraction and desorption. The optimized specifications for these variables were adopted from Sobczak et al. [29] and directly applied to the final protocol. The schematic presentation of the all-in-one thin-film microextraction protocol coupled with the LC-MS technique is presented in Figure 2 with the final parameters summarized in Figure 3.

### 2.2. Method Validation

The developed TFME-LC-MS/MS method was validated using a dedicated quantitative validation protocol designed to comply with FDA requirements [31]. The results confirmed that the proposed TFME-LC-MS/MS method provided high sensitivity and selectivity for the targeted drugs and their deuterated internal standards. In addition, good chromatographic performance was achieved for all analyzed substances, thus allowing for unambiguous qualitative and quantitative determination.

The method’s specificity was assessed by applying it to analyze blank matrices (plasma, urine, and oral fluid) from six individual donor lots. Each blank injection was processed against each LLOQ and analyzed. No peaks were detected in the extracted ion chromatograms of the negative samples at the corresponding retention times for the studied drugs.

**Figure 4:**
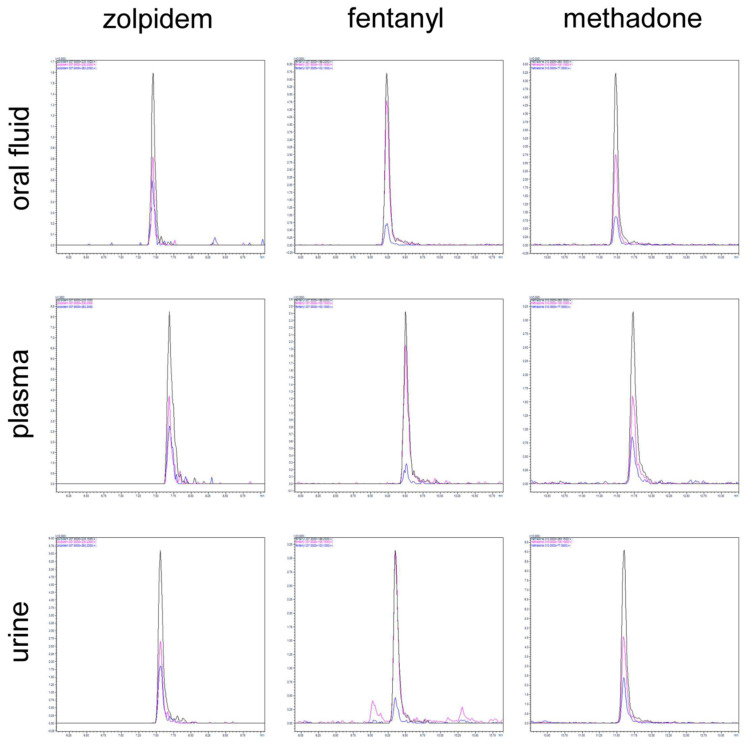
Chromatograms at lower quantification limits. Zolpidem: 1 μg L^−1^ in oral fluid; 3 μg L^−1^ in plasma and urine. Fentanyl: 3 μg L^−1^ in oral fluid; 1 μg L^−1^ in plasma and urine. Methadone: 3 μg L^−1^ in oral fluid; 1 μg L^−1^ in plasma; and 3 μg L^−1^ in urine.

Quantitative validation was conducted by using different operators to analyze various levels of the validation samples in triplicate. The results of these tests confirmed the proposed method provided satisfactory precision for all target analytes. Calibration curves were then prepared by spiking plasma, urine, and oral fluid at seven different concentrations each; determination coefficients greater than 0.971 were obtained for all compounds. Additionally, the evaluation of linearity was performed by calculating the percent deviation of a single concentration level; the evaluation of instrumental deviations within the calibration curve was also carried out according to equations presented by Mula et al. [32], thus demonstrating the method’s ability to provide satisfactory linearity in the 1–100 ng mL^−1^ range. A summary of the obtained data is presented in Table 1 and Table 2. As can be seen, the validation protocol yielded results that satisfied the acceptance criteria for all investigated parameters. Along with good results for linearity, recovery, and matrix effects, the developed protocol showed satisfactory values for intermediate precision and accuracy. The method’s satisfactory quantitative performance was further confirmed by the results for precision, which remained below 15% (intra-day: 2.7% to 9.6%; inter-day: 5.5% to 12.0%), and accuracy, which fell within the range of 80–120% (89% to 104%). The lowest concentration at which uncertainty was observed was below 20%, while signal-to-noise ratios of 3 and 10 were considered to be the LOD and LOQ, respectively. LOD values below 1 ng mL^−1^ obtained for most combinations of matrices and analytes allowed for the precise and accurate measurement of the target analytes in biological matrices that satisfied the minimum sensitivity requirements stipulated by the WADA, SAMHSA, and EWDTS. Additionally, the method’s analytical performance significantly exceeded that of routinely employed immunoassays, which have LLOQ values of 2 ng mL^−1^, 300 ng mL^−1^, and 25 ng mL^−1^ for fentanyl, methadone, and zolpidem, respectively [33]. Figure 4 presents the chromatograms at the lower quantification limits. The extraction recovery and matrix effects of the proposed sample preparation protocol were assessed using a method published elsewhere [34,35] along with the following formula:

(1) $$ME\left(\%\right)=\frac{signal_{post\;extraction\;spike}}{signal_{neat\;standard}}\times 100$$

As shown in Table 2, the optimized TFME procedure enabled satisfactory extraction recoveries and negligible matrix effects. Since the applied protocol is based on microextraction, the recoveries are dependent on the coating’s sample distribution constants and the protein-binding capacity. The experimental results show that the efficiency of matrix-compatible TFME extraction depends on the hydrophobicity of the extracted analytes and the presence of proteins in the matrix, in accordance with the basics of the “balanced coverage” method [36]. Specifically, it is assumed that matrix components present in complex matrices will significantly bind analytes, especially nonpolar compounds, thereby reducing their free concentration and, thus, their availability for extraction by the coating [36,37]. All analytes for each matrix, urine, plasma, and oral fluid in each tested condition were stable and, as shown in Table 2, remained within the acceptance criteria CV ± 15%. Ultimately, the proposed LC-MS/MS method can be considered suitable for clinical applications, as it provided excellent quantitative performance down to 1 ng mL^−1^ concentration for all studied analytes in plasma/urine/oral fluid.

### 2.3. In Silico Assessment of the TFME-LC-MS/MS Protocol’s Greenness

**Figure 5:**
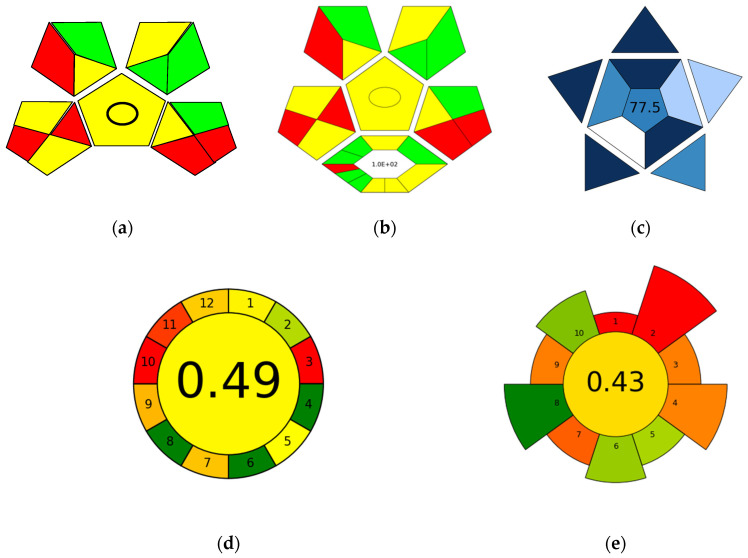
Green assessment of the TFME-LC-MS/MS protocol applying (**a**) the GAPI tool, (**b**) the ComplexGAPI tool, (**c**) the BAGI tool, (**d**) the AGREE metric, and (**e**) the AGREEprep metric.

The validation experiments confirmed that the novel, all-in-one TFME-LC-MS/MS protocol developed in this work provided good performance for the analysis of drugs of abuse in oral fluid, plasma, and urine. However, when designing a new analytical procedure, it is also critical to assess its ecological impact. Over the last decade, researchers have reported many metrics and methods for assessing the greenness of a given analytical protocol. One of the first and most cited metrics was the analytical eco-scale [38]. This metric was succeeded by more efficient metrics, including the green analytical procedure index (GAPI) [25] and the AGREE metric [26], which both featured improvements and modifications [27,28]. Most recently, the blue applicability grade index (BAGI) was developed to evaluate the practicality of a given method [39]. The main advantages of the aforementioned tools are their versatility, easy implementation, ability to assign weights, and production of a colorful and easy-to-read pictogram that displays the method’s weak and strong points. Therefore, we evaluated the proposed TFME-LC-MS/MS method’s sustainability by conducting a green assessment using a variety of metrics, software, tools, and scales, including the GAPI [25], the complementary GAPI (ComplexGAPI) [27], the BAGI [39], the AGREE [26], and the AGREE for sample preparation scale (AGREEprep) [28]. Figure 5 shows the assessment results for the validated method based on the above-listed metrics. Both the AGREE and modified GAPI tests confirmed the greenness of the proposed protocol and analytical technique. However, a deeper analysis of the results can benefit future efforts to improve the analytical method and obtain more ideal results. The GAPI considers five main items—sampling procedure, sample preparation, reagents used, and instrumentation—with the results being illustrated as individual pentagons representing 15 different areas corresponding to the environmental impact of each item (indicated using a red/green/yellow color scheme). As shown in Figure 5a,b, the GAPI and ComplexGAPI pictograms only have five red zones, which correspond to sample preparation for off-line sampling and the use of non-green solvents/reagents (lower left pentagon); waste from the instrumentation and waste treatment (bottom right corner); and safety hazards associated with the utilized reagents and solvents. Plotka-Wasylka et al. [39] recently presented a BAGI tool for evaluating an analytical method’s practicality and applicability, which identifies the method’s weak and strong points and visualizes them using asteroid pictograms, along with the respective scores. The BAGI pictogram shown in Figure 5c has only one white and two light blue zones, which correspond to the required sample volume (1 mL bioanalytical samples), the sample preparation method (SPME), and the need for sophisticated instrumentation not commonly available in most labs (LC-MS/MS). Nevertheless, the assigned BAGI score for the developed method was 77.5, which confirms its good applicability potential.

The AGREE and AGREEprep pictograms are circular in shape and divided into 12 areas corresponding to the twelve principles of green analytical chemistry. The core part of the pictogram is colored from red to green depending on the calculated AGREE value. As shown in Figure 5d,e, the AGREE and AGREEprep assessments contained only three and two dark green zones, respectively. These zones correspond to the elimination of toxic reagents (principle 11 in AGREE metric), preferably obtained from renewable sources (principle 10 in AGREE metric), proximity of the sample preparation device (principle 3 in AGREE metric) to the measurement location (in situ sampling and analysis), and compatibility with field-portable instruments, resulting in minimal time delay between the collection and analysis of the sample. Therefore, in order to improve the red zone described by principle 3 (‘if possible, measurements should be performed in situ” [26]), integrating sample preparation steps directly within the LC system might be utilized, which streamlines the entire process, reduces manual handling, and enhances overall efficiency. Alternatively, SPME offers unique opportunities for on-site sampling and screening [40] using a portable MS system and/or direct coupling to MS [41], which constitutes a valid alternative to more labor- and solvent-intensive liquid chromatography methods. In turn, red zones that arose in the case of principle 10 (‘sustainable and renewable reagents are preferred’ [26]) and principle 11 (‘Safer reagents less toxic, hazardous are preferred’ [26]) are due to the use of strong and highly hazardous organic solvents for TFME device preparation processes (e.g., *N*,*N*-dimethylformamide (DMF) or concentrated hydrochloric acid) and the consumption of organic solvents (e.g., acetonitrile and methanol) typically required in the postprocessing and functionalization of sample preparation devices (e.g., for stationary phase immobilization) [42]. Most recently, a biocompatible 3D-printed TFME polyamide noncoated device for adsorption-based microextraction (known as PANDA extraction) [30] was introduced to fulfill the principles of green chemistry, demonstrated in principles 10 and 11 of the AGREE metric [26]. Therefore, using 3D printing technology for the preparation of microextraction devices presents clear advantages associated with the use of prototype 3D-printed devices composed of a ready-to-use stationary phase made of polyamide blends with extraction capacities [30].

## 3. Materials and Methods

### 3.1. Human Matrices

Method validation was performed using drug-spiked matrices (pooled from multiple donors or obtained from single donors) according to the appropriate guidelines. Drug-free human oral fluid, urine, and plasma were supplied by Lee Biosolutions^TM^ Inc. (Maryland Heights, MO, USA). In addition, one of the authors volunteered to donate oral fluid and urine specimens.

### 3.2. Chemicals

All experiments were performed using the following LC-MS-grade solvents and additives: acetonitrile (CHROMASOLV^TM^, Honeywell International Inc., Charlotte, NC, USA); formic acid (Optima^TM^, Fisher Chemical, Thermo Fisher Scientific Inc., Waltham, MA, USA); methanol (CHROMASOLV^TM^, Honeywell International Inc., Charlotte, NC, USA); and water (LiChrosolv^®^, Merck KgaA, Darmstadt, Germany).

The TFME coatings were prepared using concentrated hydrochloric acid (Fluka^TM^, Honeywell International Inc., Charlotte, NC, USA), *N*,*N*-dimethylformamide (Sigma-Aldrich^®^, Merck Group, Darmstadt, German), polyacrylonitrile (Aldrich^®^, Merck KgaA, Darmstadt, Germany), 10 μm silica particles bonded with octyl (Luna^®^ C8(2), Phenomenex^®^ Inc., Torrance, CA, USA), and 3-cyanopropyl (Luna^®^ CN, Phenomenex^®^ Inc., Torrance, CA, USA) ligands.

Certified reference materials were purchased as 1 g L^−1^ stock solutions in methanol. Fentanyl and zolpidem (tartrate) were purchased from LGC Standards (LGC Poland, Łomianki, Poland), and (±) methadone was purchased from Cerillant^®^ (Merck Life Science Sp.z.o.o, Poznań, Poland). Deuterium-labeled reference materials of fentanyl D5, (±)-methadone D3, and zolpidem D6 were acquired from Cerillant^®^ (Merck Life Science Sp.z.o.o, Poznań, Poland) as 100 mg L^−1^ stock solutions in methanol.

### 3.3. Working/Standard Solutions and Calibrators

The stock solutions of certified materials (1 g L^−1^ in methanol) were stored at −20 °C in the dark until analysis, with standard working solutions being prepared daily in methanol. Calibrators were prepared for at least seven concentration levels between 1 and 100 ng mL^−1^ by spiking pooled samples of the blank plasma, urine, or oral fluid, while quality control samples were prepared in methanol at 1, 3, 40, and 100 ng mL^−1^ by fortifying the blank plasma, urine, or oral fluid. The QC samples were analyzed alongside each batch of matrix samples to assess the method’s intra- and inter-day precision and accuracy. All prepared solutions were stored at 4 °C prior to LC-MS/MS analysis.

### 3.4. Preparation of the Thin-Film Microextraction Blades

The metal blades (12-pin) used as supports for the TFME coatings were purchased from PAS Technology Deutschland GmbH (Magdala, Germany). The supports were etched in concentrated hydrochloric acid for 60 min in an ultrasonic bath, then cleaned with distilled water and dried in an oven at 150 °C for 30 min.

The coating slurry was prepared by dispersing the particles in a DMF solution of PAN. The same proportions were used for each type of coating (i.e., PAN/DMF/particles (1.000:18.380:2.375, m/m/m)). All particles—namely, octadecyl, octyl, phenyl-hexyl, cyanopropyl, benzenesulfonic acid, and unbounded silica—were supplied by Phenomenex (Torrance, CA, USA). The characteristics of the particles used to prepare the stationary phases are reported elsewhere in the literature [29]. The coatings were applied at a length of one centimeter via a spraying method, according to the previously published instructions [42]. In total, ten layers of coating were applied, with each layer being dried at 110 °C for 3 min after application (to prevent thermal degradation of some particles observed at 180 °C [43]).

### 3.5. Sample Preparation and Extraction Protocol

The samples were prepared by spiking drug-free matrices with reference standards to the required concentrations, followed by mixing on a benchtop shaker (850 min^−1^ agitation) for 60 min at room temperature (ca. 20 °C) to allow drug–protein binding. For oral fluid only, the drug-spiked samples were aliquoted to oral fluid collection swabs (8 × 40 mm), which had been gifted by the Porex^®^ Filtration Group (St. Charles, MI, USA). The swabs were then centrifuged (1500× *g*, 3 min, 4 °C) in special tubes featuring a pre-cut hole in the bottom of the inner tube (Salivette^®^, Sarstedt AG & Co. KG, Nümbrecht, Germany) to separate the liquid from the swabs.

All extractions were performed using 1 mL of sample in 96-well DeepWell^TM^ Plates (Nunc^TM^, Thermo Fisher Scientific Inc., Waltham, MA, USA) with an SH10 Heater-Shaker (Ingenieurbüro CAT, M. Zipperer GmbH, Ballrechten-Dottingen, Germany). This semi-automated setup allowed up to 192 samples to be processed simultaneously, thus achieving a high sample throughput of approximately 3 min per sample. The extractions were performed in an air-conditioned laboratory with the temperature controlled to approximately 20.0 °C, and the samples were spiked with isotope-labelled internal standards at 3 ng mL. LC-MS-grade formic acid (Optima^TM^, Thermo Fisher Scientific Inc., USA), methanol (CHROMASOLV^TM^, Honeywell International Inc.,Charlotte, NC, USA), and water (LiChrosolv®, Merck KgaA, Darmstadt, Germany) were used in all of the above steps. The full extraction protocol is presented in Figure 4.

### 3.6. LC-MS/MS Method

The LC-MS/MS system consisted of a Shimadzu Nexera UHPLC system equipped with a binary solvent pump, a heated column compartment, a cooled autosampler, and a triple quadrupole mass spectrometry detector (LCMS-8060, Shimadzu Corporation, Kyoto, Japan). Chromatographic separations were carried out using an Agilent InfinityLab Poroshell (120 EC-C18, Agilent, Santa Clara, CA, USA) analytical column (3 mm × 100 mm, 2.7 µm) fitted with a guard column (3 mm × 5 mm, 2.7 µm) and maintained at 25 °C and 4 °C for the column and sample temperature, respectively. Mobile phase (A) consisted of water with 0.1% formic acid, while mobile phase (B) consisted of acetonitrile with 0.1% formic acid and was applied successfully for reversed-phase gradient elution prior to the separation of the same solutes [29,30]. The LC process used 0.8 µL of extract (in acetonitrile/water/formic acid (80/19.9/0.1, *v*/*v*/*v*)) diluted with 3.2 µL of water for a total injection volume of 4.0 µL per sample. The dilution was automatically performed by the autosampler. The resultant chromatograms and retention times, along with the precursor–product ion transitions, are presented in the Supplementary Materials (Figure S1 and Table S1, respectively).

MS/MS analysis was performed on a triple quadrupole mass spectrometry detector (LCMS-8060, Shimadzu Corporation, Kyoto, Japan), which was connected to the UHPLC system via an electrospray ionization (ESI) interface. The ESI MS/MS parameters were set as follows: nebulizing gas flow, 3 L min^−1^; heating gas flow, 10 L min^−1^; interface temperature, 300 °C; DL temperature, 250 °C; heat block temperature, 400 °C; and drying gas flow, 10 L min^−1^. Nitrogen was used as the nebulizing and heating gas, and argon (99.9% purity) was used as the collision gas. The separated analytes were introduced into the LCMS-8060 mass spectrometer equipped with ESI in positive ion mode (ESI+) for quantification. Detection was obtained in multiple reaction monitoring (MRM) mode, including three product ions. Two of the MRMs confirming the analytes are presented are shown in the Supporting Information (Table S1). Data acquisition and processing were performed using LCMS LabSolution software v.5.80 from Shimadzu, and the statistical calculations were conducted using Excel for Windows.

### 3.7. Validation

The proposed method was validated in accordance with the criteria suggested by the US FDA’s Guidance for Industry-Bioanalytical Method Validation 19 [31]. Parameters such as linearity, selectivity, precision, extraction efficiency, limits of detection (LOD), limits of quantification (LOQ), and the matrix effects of the studied analytes (i.e., fentanyl, methadone, and zolpidem) were validated separately for each matrix. The method’s specificity was evaluated by comparing chromatograms of blank matrices (plasma, urine, or oral fluid) collected from at least six different drug-free donors for interference at the retention times of fentanyl, methadone, and zolpidem and their ISs.

Calibration curves were built for matrices (blood, urine, and oral fluid) by plotting the peak ratios of fentanyl/methadone/zolpidem to the IS against the nominal concentrations of the calibration standards at 1, 3, 10, 15, 20, 25, 30, 40, 50, and 100 ng mL^−1^. Measurements were taken in triplicate for each concentration and used to create the calibration curve for each sample batch. The linear least-squares regression of the calibration lines, slopes, intercepts, and correlation coefficients were obtained from the peak area ratios of fentanyl/methadone/zolpidem to the IS versus the corresponding concentrations. The limit of detection (LOD) and limit of quantification (LOQ) were described as the lowest concentration with signal-to-noise ratios of 3 and 10, respectively. For the determination of the LOQ, satisfactory accuracies of 80–120% and precisions within 20% were evaluated and confirmed using six replicate analyses.

QC samples at 4 various concentrations (LOD: 1 ng mL^−1^; LOQ: ng mL^−1^; MID: 30–50% of curve range, here 30 ng mL^−1^; and HIGH: 75–100% of curve range, here 100 ng mL^−1^) were used to assess the method’s intra- and inter-day (three different days experiment’s) precision. Each batch consisted of a matrix blank, specified concentrations of calibration standards, and different QC samples. SST samples were run also to check the stability of the instrument and the repeatability retention time. Finally, carry-over was evaluated by analyzing blank matrix samples immediately after testing triplicates of high-concentration control samples (100 ng mL^−1^).

The samples for the stability studies were kept at specific conditions for a defined time prior to the extractions. The evaluated conditions included 24 h at room temperature; 5 days in a refrigerator (4 °C); and 3 freeze/thaw cycles (−20 °C/+20 °C). In addition, the extracts in desorption solvent (acetonitrile/water/formic acid (80.0/19.9/0.1, *v*/*v*/*v*)) were kept for 6.5 days at 4 °C to assess their stability in a refrigerated autosampler.

## 4. Conclusions

A fit-for-purpose TFME-LC-MS/MS protocol was developed and validated for the detection of zolpidem, methadone, and fentanyl in urine, plasma, and oral fluid samples. To the best of our knowledge, this is the first report to detail an all-in-one SPME-based sample preparation protocol that provides good selectivity, sensitivity, and repeatability for the detection of popular drugs of abuse in three different matrices. Furthermore, the proposed TFME-LC-MS/MS method can be adopted in any laboratory, including WADA, EWDTS, or SAMHSA, as no carry-over was observed after an optional cleaning procedure. Additionally, the list of potential substances can be extended, as microextraction is equilibrium-based and does not require exhaustive extraction; thus, once the parameters have been optimized and validated, this method can be applied for the analysis of a wide range of small-molecule compounds. Given the worldwide increase in drug abuse, particularly polyconsumption and overdose rates for zolpidem, methadone, and fentanyl, drug monitoring using the proposed method can provide accurate information regarding drug abuse, which could prove invaluable in preventing and combating drug-related crimes. Furthermore, the application of the proposed protocol can also provide important technical support for the comprehensive implementation of all narcotics control work.

The greenness of the proposed TFME-LC-MS/MS method was quantitatively analyzed using different evaluation approaches, particularly with respect to the consumed solvents/reagents, its energy consumption, occupational hazards, and the amount of waste that is generated. Although the proposed protocol has the potential to supplement or replace conventional sample preparation techniques, efforts should be directed to the treatment of waste and safety hazards related to the reagents and solvents used. Ideally, this will be achieved by (1) replacing traditional solvents with greener alternatives and (2) moving to direct analysis and remote sensing.

## Figures and Tables

**Table 1:** Calibration curve parameters.

Matrix	Drugs of Abuse	Linearity Range ng mL^−1^	R^2^	Slope (m)	Intercept (q)	$\Delta \bar{RF}\%$	LOD ng mL^−1^	LOQ ng mL^−1^
Urine	Zolpidem	3–100	0.971	62,645	28,338	11.44	1	3
	Fentanyl	1–100	0.991	97,278	70,241	8.00	0.4	1
	Methadone	3–100	0.998	126,872	−8901	5.44	1	3
Plasma	Zolpidem	3–100	0.996	19,998	−17,490	12.13	1	3
	Fentanyl	1–100	0.996	104,063	−9263	6.67	0.4	1
	Methadone	1–100	0.998	169,015	−59,173	6.78	0.4	1
Oral fluid	Zolpidem	1–100	0.995	46,848	−4588	9.13	0.3	1
	Fentanyl	3–100	0.995	74,614	−51,954	5.01	1	3
	Methadone	3–100	0.998	75,469	−16,221	3.89	1	3

**Table 2:** Summary of validation parameters.

Matrix	Drugs of Abuse	Matrix Effect (%)	Extraction Efficiency (%)	Precision Intra-Day (%) QC-LOQ	Precision Inter-Day (%) QC-LOQ	Accuracy (%)	Stability			
							RT (%)	F/T (%)	FR (%)	AS (%)
Urine	Zolpidem	97.4	42.8	7.4	6.2	89	9	7	11	2
	Fentanyl	96.0	62.7	4.9	5.5	99	10	11	4	3
	Methadone	96.0	76.2	4.0	8.8	104	9	5	3	0
Plasma	Zolpidem	93.6	34.3	8.3	7.8	102	−13	−11	15	−9
	Fentanyl	93.3	34.5	4.3	8.1	104	5	4	13	−1
	Methadone	92.2	62.4	4.7	11.1	99	5	2	15	−2
Oral fluid	Zolpidem	85.5	26.7	9.6	11.8	103	−15	7	7	15
	Fentanyl	87.4	38.0	4.8	12.0	101	−5	3	3	−9
	Methadone	87.7	36.7	2.7	12.0	102	−9	−11	9	−5

RT: stability for 24 h at room temperature; F/T: 3 freeze/thaw cycles (−20 °C/+20 °C); FR: 5 days at refrigerator (4 °C); AS: extracts in desorption solvent (acetonitrile/water/formic acid (80.0/19.9/0.1, *v/v/v*)) for 6.5 days at 4 °C.

## Data Availability

Data is contained within the article or supplementary material.

## Supplementary Materials

The following supporting information can be downloaded at: https://www.mdpi.com/article/10.3390/molecules29020335/s1, Figure S1: Opioid overdose deaths over the past two decades. The figure illustrates three “waves” of the opioid epidemic, with synthetic opioids, primarily fentanyl and its analogs, driving the “third wave.”; Figure S2: Chromatogram of the drug-spiked urine extract; Table S1: LC-MS/MS parameters for the analyzed substances.
