# Supplementary material for: Developing and Evaluating the Greenness of a Reliable, All-in-One Thin-Film Microextraction Protocol for Determining Fentanyl, Methadone, and Zolpidem in Plasma, Urine, and Oral Fluid
Source: Molecules. 2024 Jan 9;29(2):335. doi: 10.3390/molecules29020335 (PMC10818652; doi:10.3390/molecules29020335)
Supplement: Supplementary file 1 [file molecules-29-00335-s001.zip › molecules-2763312-supplementary.pdf]

Supplementary Materials

Developing and Evaluating the Greenness of a Reliable,
All-in-One Thin-Film Microextraction Protocol for Determining Fentanyl, Methadone, and Zolpidem in Plasma, Urine, and Oral Fluid

Krzysztof Goryński [1,*], Łukasz Sobczak [2,†] and Dominika Kołodziej [2,†]

[1] Faculty of Chemical Technology and Engineering, Bydgoszcz University of Science and Technology, Seminaryjna 3, 85-326 Bydgoszcz, Poland
[2] Faculty of Pharmacy, Nicolaus Copernicus University in Toruń, Jurasza 2, 85-089 Bydgoszcz, Poland
* Correspondence: gorynski@pbs.edu.pl
† These authors contributed equally to this work.

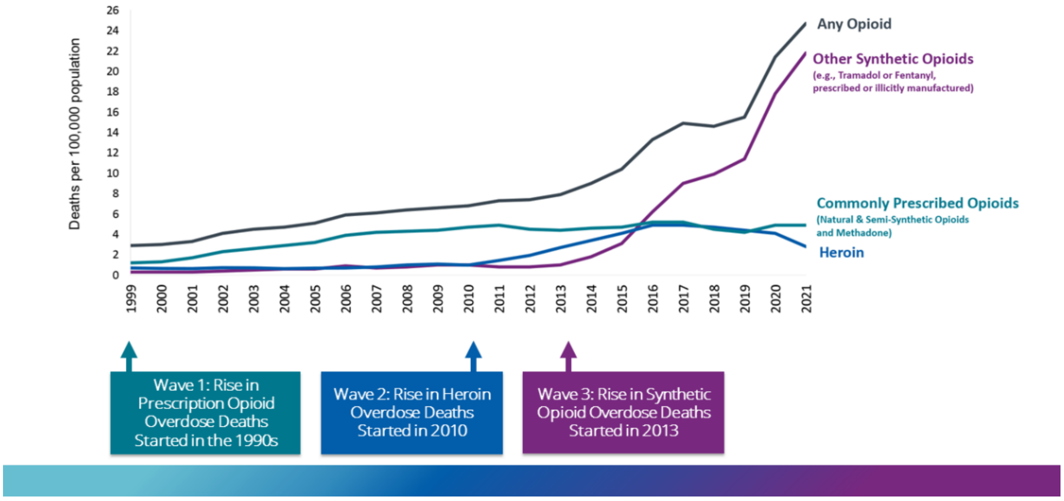

Figure S1. Opioid overdose deaths over the past two decades. The figure illustrates three "waves" of the opioid epidemic, with synthetic opioids, primarily fentanyl and its analogs, driving the "third wave." This figure (modified) is taken from the National Vital Statistics SystemcMortality File (https://www.cdc.gov/drugoverdose/resources/graphics/images/overdose/3-wave-opioid-overdose-death-line-graph.png).

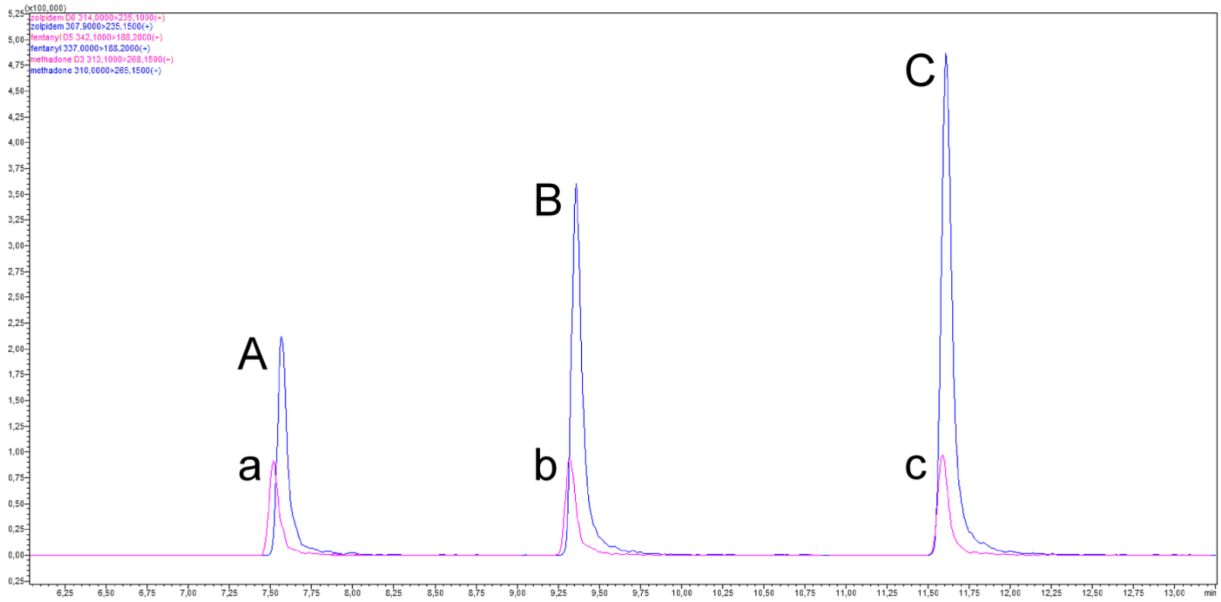

Figure S2. Chromatogram of the drug-spiked urine extract.

Analytes at 20 µg L^{-1} concentration: A – zolpidem; B – fentanyl; and C – methadone. Internal standards at 3 µg L^{-1} concentration: a – zolpidem D_6; b – fentanyl D_5; and c – methadone D_3.

Table S1. LC-MS/MS parameters for the analyzed substances.

Substance	Retention Time [min]	Precursor Ion [m/z]	Product Ions [m/z]		
zolpidem D_6	7.517	314.00	235.10	236.20	263.20
zolpidem	7.573	307.90	235.15	236.20	263.20
fentanyl D_5	9.325	342.10	188.20	105.10	103.05
fentanyl	9.364	337.00	188.20	105.15	103.10
methadone D_3	11.585	313.10	268.15	105.05	77.15
methadone	11.606	310.00	265.15	105.10	77.05
